# Rapid quantification of fatty acids in plant oils and biological samples by LC-MS

**DOI:** 10.1007/s00216-021-03525-y

**Published:** 2021-07-22

**Authors:** Elisabeth Koch, Michelle Wiebel, Carolin Hopmann, Nadja Kampschulte, Nils Helge Schebb

**Affiliations:** Chair of Food Chemistry, Faculty of Mathematics and Natural Sciences, University of Wuppertal, Gaussstrasse 20, 42119 Wuppertal, Germany

**Keywords:** Chromatographic separation, *Pseudo*-SRM, Non-esterified fatty acids, Saponification, Oxylipins

## Abstract

Analysis of fatty acids (FA) in food and biological samples such as blood is indispensable in modern life sciences. We developed a rapid, sensitive and comprehensive method for the quantification of 41 saturated and unsaturated fatty acids by means of LC-MS. Optimized chromatographic separation of isobaric analytes was carried out on a C8 reversed phase analytical column (100 × 2.1 mm, 2.6 μm core–shell particle) with a total run time of 15 min with back pressure lower than 300 bar. On an old triple quadrupole instrument (3200, AB Sciex), *pseudo* selected reaction monitoring mode was used for quantification of the poorly fragmenting FA, yielding limits of detection of 5–100 nM. Sample preparation was carried out by removal of phospholipids and triglycerides by solid-phase extraction (non-esterified fatty acids in oils) or saponification in *iso*-propanol (fatty acyls). This is not only a rapid strategy for quantification of fatty acyls, but allows the direct combination with the LC-MS-based analysis of fatty acid oxidation products (eicosanoids and other oxylipins) from the same sample. The concentrations of fatty acyls determined by means of LC-MS were consistent with those from GC-FID analysis demonstrating the accuracy of the developed method. Moreover, the method shows high precisions with a low intra-day (≤ 10% for almost all fatty acids in plasma and ≤ 15% in oils) and inter-day as well as inter-operator variability (< 20%). The method was successfully applied on human plasma and edible oils. The possibility to quantify non-esterified fatty acids in samples containing an excess of triacylglycerols and phospholipids is a major strength of the described approach allowing to gain new insights in the composition of biological samples.

**Graphical abstract:**

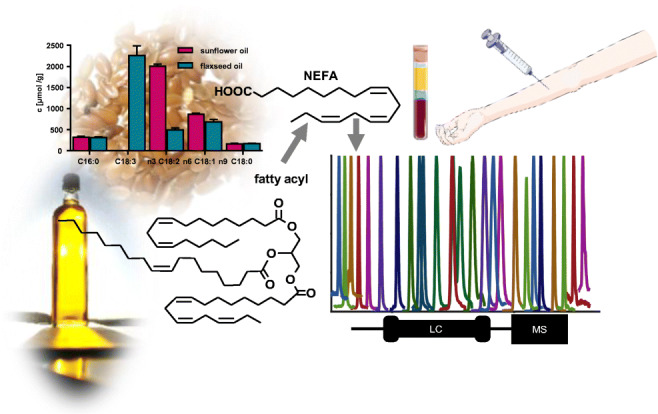

**Supplementary Information:**

The online version contains supplementary material available at 10.1007/s00216-021-03525-y.

## Introduction

Fatty acids play a fundamental role in the biology of living organisms, e.g., by influencing properties of biomembranes, storing and providing energy, or being involved in cell signaling [1–3]. Especially long-chain polyunsaturated fatty acids (PUFA) such as arachidonic acid (ARA), eicosapentaenoic acid (EPA) or docosahexaenoic acid (DHA) are involved in many (patho)physiological processes, i.a., through their oxidation products. These eicosanoids and other oxylipins are highly potent lipid mediators regulating for example inflammation, vasoconstriction or pain [4, 5]. Dietary supplementation of n3-PUFAs or reducing the intake of n6-PUFA is a promising way to modulate endogenous fatty acid distribution which has been demonstrated in numerous intervention studies [6–8].

The basic structure of fatty acids is a linear hydrocarbon chain with a varying number of double bonds. A large number of structurally similar molecules, e.g., n3- vs. n6-PUFA, leads to challenges in analytics. Fatty acyls are often analyzed by means of gas chromatography coupled to flame ionization detection (GC-FID) or mass spectrometry (GC-MS) due to the high separation efficiency and good sensitivity of GC [9–11]. However, transesterification or derivatization is necessary for this analytical procedure, which is on the one hand laborious and time-consuming and on the other hand can lead to discrimination of analytes [12]. Similarly, the need for derivatization makes quantifying non-esterified fatty acids (NEFA) in biological samples by GC challenging due to the high amount of fatty acyls occurring in different lipid classes such as triacylglycerols or phospholipids. Fractionation of these lipid classes by solid-phase extraction (SPE) is a powerful tool to separate NEFA from other lipid species [13]: However, already 1–2% of unremoved triacylglycerols or phospholipids disturbs the quantification of low abundant NEFA in biological samples and plant oils.

The use of liquid chromatography-mass spectrometry (LC-MS) has raised strong interest in fatty acid analysis in recent years. Even though some published methods include derivatization of the fatty acids, e.g., to improve ionization efficiency [14, 15], determination by LC-MS offers the possibility of analyzing fatty acids directly [16, 17]. Reversed phase columns based on modified silica gel are commonly used as stationary phase usually in combination with more non-polar solvents such as *iso*-propanol/acetonitrile mixtures [16, 18].

However, none of the published LC-MS approaches [15, 16, 19–25] fulfills our needs regarding comprehensiveness, chromatographic separation of isobaric fatty acids, high sample throughput and applicability for a wide range of matrices. Therefore, we developed herein a new method which (i) covers a comprehensive set of biologically occurring fatty acids, (ii) allows rapid analysis (< 15 min) but separation of positional isomers, and (iii) requires only simple sample preparation by saponification following dilution in organic solvent. The latter allows us to analyze the oxidation products of fatty acids, eicosanoids and other oxylipins, from the same sample preparation using an established targeted oxylipin metabolomics method [26]. The method optimization and performance were characterized and compared to earlier published methods and the accuracy was demonstrated by cross-validation with a standard GC-FID approach. Finally, the method was successfully applied to analyze human plasma and refined as well as virgin plant oils.

## Material and methods

### Chemicals and biological materials

Fatty acid standards (C6:0, C7:0, C8:0, C9:0, C10:0, C11:0, C12:0, C13:0, C15:0, C17:0, C18:2 n6, C18:1 n9, C18:0, C20:0, C21:0, C8:0-d15, C12:0-d23, and C20:5 n3-d5) were purchased from Merck (Darmstadt, Germany). The internal standards C16:0-d4 and C18:0-d5 were bought from Eurisotop (Saarbrücken, Germany). All other fatty acid standards were purchased from Cayman Chemicals (Ann Arbor, MI, USA). Fatty acid methyl ester (FAME) standards for the FAME reference mix were from Restek (marine fish oil mix; Bad Homburg vor der Höhe, Germany), Merck (FAME C22:4 n6, FAME C22:5 n3, FAME C25:0, Supelco 37 Component FAME Mix; Darmstadt, Germany), Fluka/Honeywell (FAME C19:0; Offenbach, Germany), and Cayman Chemicals (FAME C18:4 n3, FAME C20:3 n9, FAME C20:4 n3; Ann Arbor, MI, USA). The used edible oils (refined and virgin sunflower oil as well as virgin flaxseed oil) samples were bought in local supermarkets in Wuppertal, Germany. Pooled human EDTA plasma was generated from healthy individuals as described [26] in accordance with the guidelines of the Declaration of Helsinki and approved by the ethics committee of the University of Wuppertal. Acetonitrile (ACN) and ethanol (EtOH) were obtained from VWR (Darmstadt, Germany) and methanol (MeOH), *iso*-propanol, as well as acetic acid (HAc) from Fisher Scientific (Schwerte, Germany). Ultra-pure water was generated using the Barnstead Genpure Pro system from Thermo Fisher Scientific (Langenselbold, Germany). All other chemicals were from Merck (Darmstadt, Germany).

### LC-ESI(−)-MS analysis

Analysis was carried out on a 1260 Infinity LC System (Agilent, Waldbronn, Germany) coupled to a API 3200 instrument (AB Sciex, Darmstadt, Germany). Ionization was carried out in negative electrospray ionization (ESI(−)) mode with the following source settings: ion spray voltage − 4500 V, curtain gas (nitrogen, N_2_-generator NGM 33, cmc Instruments, Eschborn, Germany) 35 psi, nebulizer gas (gas 1, purified compressed air; “zero air”) 70 psi generated with a RAMS 05ZA (cmc Instruments, Eschborn, Germany), drying gas (gas 2, purified compressed air) 55 psi, temperature 500 °C. The sprayer offset was 0.511 cm for the vertical and 0.519 cm for the horizontal axis. The electrode protrusion was approximately 1 mm. Ten microliters of samples were injected by an HTC PAL autosampler (CTC Analytics, Switzerland, local distributor: Axel Semrau, Sprockhövel, Germany) equipped with a 25-μl syringe and a 20-μl sample loop. Samples were cooled at 4 °C. Separation of fatty acids was carried out on Kinetex C8 core–shell reversed phase column (100 × 2.1 mm, particle size 2.6 μm, pore size 10 nm; Phenomenex, Aschaffenburg, Germany) kept at 40 °C. The analytical column was equipped with an inline filter (0.3 μm, 1290 infinity II inline filter, Agilent, Waldbronn, Germany) and a SecurityGuard Ultra C8 cartridge as precolumn (2.1 mm ID, Phenomenex, Aschaffenburg, Germany). Solvent B of the mobile phase consisted of ACN/MeOH/HAc (80/15/0.1, *v*/*v*/*v*) and solvent A was 0.1% HAc mixed with 5% of solvent B. The following linear gradient was used: 0.0–1.0 min isocratic 20% B, 1.0–1.5 min linear from 20% B to 66% B, 1.5–8.0 min isocratic 66% B, 8.0–11.0 min linear from 66% B to 100% B, 11.0–14.0 min isocratic 100% B, 14.0–14.5 min linear from 100% B to 20% B followed by equilibration for 0.5 min. This resulted in a total run time of 15 min. The Analyst software (version 1.6.2, Sciex) was used for instrument control as well as data acquisition and Multiquant (version 2.1.1, Sciex) for peak integration and quantification.

### Calibration and quantification

Stock solutions of the individual fatty acids were mixed and diluted in EtOH using glass volumetric flasks to concentration levels of 0.05, 0.075, 0.1, 0.25, 0.5, 0.75, 1.0, and 2.5 μM. For fatty acids which often occur in high concentrations in biological samples (C16:0, C16:1 n7, C18:0, C18:1 n9, C18:2 n6, C20:4 n6) final concentration levels were 0.1, 0.25, 0.5, 1.0, 2.5, 5.0, 10.0, 15.0, and 20.0 μM. Concentrations of fatty acids in stock solution (200 μM) were verified by GC-FID following HCl-catalyzed transmethylation to fatty acid methyl esters (FAME) according to Ostermann et al. (see Supplementary Information (ESM) Fig. S1) [12]. If the determined concentration of a fatty acid in stock solution was not within ± 15%, a correction factor was used. Additionally, the concentration of PUFAs in the calibration solution is monitored using a FAME reference mix to compensate for autoxidative degradation. The FAME reference mix was directly analyzed by GC-FID and prepared as sample for LC-MS determination. If the PUFA concentration determined by GC-FID and LC-MS was not within ± 10%, a second correction factor was used. C8:0-d15, C12:0-d23, C16:0-d4, C18:0-d5, C18:1 n9-d17, C18:2 n6-d4, C20:0-d3, C20:3 n6-d6, C20:4 n6-d8, C20:5 n3-d5, and C22:6 n3-d5 were used as internal standards at a concentration of 0.2 μM.

For calibration, the peak area ratios (analyte/IS) were plotted against the concentration ratio (analyte/IS). Calibration curves were calculated using linear or quadratic least square regression (weighting: 1/*x*^2^, Table 1). The limit of detection (LOD) was determined by a signal-to-noise ratio of ≥ 3 and the lower limit of quantification (LLOQ) by signal-to-noise ratio of ≥ 5 and accuracy of ± 20% within the calibration curve. For fatty acids which are ubiquitously detectable in blank injection, i.e., injection of EtOH, the LLOQ was set to the concentration yielding a peak height of at least twofold of the peak height in blank injections and accuracy within the calibration curve of ± 20%.

**Table 1:** LC-ESI(−)-MS/MS parameters and performance for the quantification of fatty acids. Shown are all fatty acids covered by the method, their mass transitions for quantification in scheduled SRM mode, specific electronic MS parameters (declustering potential (DP), collision energy (CE)), their internal standards (IS), retention time (*t*_R_), full peak width at half maximum (FWHM), the calibration range, the limit of detection (LOD) and the lower limit of quantification (LLOQ). Scheduled selected reaction monitoring mode (SRM) using nitrogen as collision gas (12 psi) with a detection window of ± 35 s around the expected retention time was used for analyte detection

**Analyte**	**Mass transition**		**Electronic parameters**		**Internal standard**	***t***_**R**_^**a**^	**FWHM**^**b**^	**Calibration range**			**LOD**		**LLOQ**
	**Q1**	**Q3**	**DP**	**CE**		**(min)**	**(s)**	**(μM)**			**(μM)**	**ng in column**	**(μM)**
			**(v)**										
C6:0	115.2	115.2	− 24	− 14	C8:0-d15	2.81 ± 0.01	3.4 ± 0.1	0.1	–	2.5	–^c^	–^c^	0.1^e^
C7:0	129.2	129.2	− 42	− 10	C8:0-d15	3.05 ± 0.01	3.2 ± 0.1	0.075	–	2.5	–^c^	–^c^	0.075^e^
C8:0	143.2	143.2	− 32	− 10	C8:0-d15	3.30 ± 0.01	3.2 ± 0.2	0.1	–	2.5	–^c^	–^c^	0.1^e^
C9:0	157.1	157.1	− 42	− 14	C8:0-d15	3.61 ± 0.01	3.3 ± 0.1	−^d^		–	–^c^	–^c^	−^d^
C10:0	171.2	171.2	− 44	− 14	C8:0-d15	4.01 ± 0.01	3.5 ± 0.2	0.05	–	2.5	–^c^	–^c^	0.05^e^
C11:0	185.1	185.1	− 36	− 16	C12:0-d23	4.52 ± 0.01	3.8 ± 0.1	0.05	–	2.5	–^c^	–^c^	0.05^e^
C12:0	199.1	199.1	− 44	− 14	C12:0-d23	5.19 ± 0.02	4.5 ± 0.2	0.1	–	2.5	–^c^	–^c^	0.1^e^
C13:0	213.2	213.2	− 46	− 16	C12:0-d23	6.10 ± 0.03	5.8 ± 0.3	0.05	–	2.5	0.005	0.01	0.01
C14:1 n5	225.2	225.2	− 46	− 16	C12:0-d23	5.74 ± 0.03	5.3 ± 0.2	0.05	–	2.5	0.005	0.01	0.01
C14:0	227.1	227.1	− 46	− 14	C12:0-d23	7.32 ± 0.04	7.7 ± 0.5	0.075	–	2.5	–^c^	–^c^	0.075^e^
C15:1 n5	239.2	239.2	− 36	− 14	C12:0-d23	6.79 ± 0.04	7.0 ± 0.3	0.05	–	2.5	0.006	0.01	0.012
C15:0	241.3	241.3	− 44	− 14	C12:0-d23	9.00 ± 0.06	11.4 ± 0.6	0.05	–	2.5	0.01	0.02	0.025
C16:1 n7	253.3	253.3	− 48	− 20	C18:1 n9-d17	8.28 ± 0.05	9.4 ± 0.4	0.1	–	20	0.005	0.01	0.01
C16:0	255.2	255.2	− 44	− 20	C16:0-d4	10.38 ± 0.03	6.8 ± 0.4	0.5	–	20	–^c^	–^c^	0.5^e^
C17:0	269.3	269.3	− 48	− 20	C20:0-d3	11.03 ± 0.02	4.1 ± 0.3	0.05	–	2.5	0.005	0.01	0.01
C18:4 n3	275.3	275.3	− 36	− 16	C20:5 n3-d5^f^	6.25 ± 0.03	6.0 ± 0.2	0.05	–	2.5	0.01	0.03	0.025
	275.3	231.3	− 36	− 16		6.25 ± 0.03	5.9 ± 0.5	0.075	–	2.5	0.05	0.1	0.075
C18:3 n6	277.2	277.2	− 46	− 22	C20:5 n3-d5	7.75 ± 0.05	8.3 ± 0.6	0.05	–	2.5	0.025	0.07	0.05
C18:3 n3	277.2	277.2	− 44	− 24	C20:5 n3-d5	7.46 ± 0.04	7.9 ± 0.4	0.05	–	2.5	0.025	0.07	0.05
C18:2 n6	279.3	279.3	− 46	− 16	C18:2 n6-d4	9.52 ± 0.06	9.6 ± 0.3	0.1	–	15	–^c^	–^c^	0.01^e^
C18:1 n9	281.4	281.4	− 46	− 18	C18:1 n9-d17	10.80 ± 0.02	4.7 ± 0.2	0.1	–	15^g^	–^c^	–^c^	0.05^e^
C18:0	283.2	283.2	− 46	− 20	C18:0-d5	11.47 ± 0.01	5.5 ± 0.5	1	–	20	–^c^	–^c^	1.0^e^
C19:0	297.4	297.4	− 46	− 20	C20:0-d3	11.79 ± 0.01	3.4 ± 0.3	0.05	–	2.5	0.005	0.01	0.01
C20:5 n3	301.2	301.2	− 46	− 16	C20:5 n3-d5^f^	7.43 ± 0.05	7.8 ± 0.4	0.05	–	2.5	0.025	0.08	0.05
	301.2	257.2	− 46	− 16		7.43 ± 0.05	7.6 ± 0.6	0.075	–	2.5	0.05	0.2	0.075
C20:4 n6	303.2	303.2	− 46	− 18	C20:4 n6-d8^f^	9.43 ± 0.06	9.8 ± 0.5	0.1	–	20	0.02	0.06	0.05
	303.2	259.2	− 46	− 18		9.43 ± 0.06	9.1 ± 0.9	0.1	–	20	0.05	0.2	0.1
C20:4 n3	303.3	303.3	− 46	− 18	C20:5 n3-d5^f^	8.74 ± 0.06	10.0 ± 0.5	0.05	–	2.5	0.01	0.03	0.025
	303.3	259.2	− 46	− 18		8.75 ± 0.05	9.4 ± 1.0	0.075	–	2.5	0.05	0.2	0.075
C20:3 n9	305.4	305.4	− 46	− 14	C22:6 n3-d5	10.69 ± 0.02	4.5 ± 0.1	0.05	–	2.5	0.006	0.02	0.013
C20:3 n6	305.4	305.4	− 46	− 14	C20:3 n6-d6	10.37 ± 0.03	5.5 ± 0.1	0.05	–	2.5	0.005	0.02	0.01
C20:2 n6	307.3	307.3	− 48	− 24	C22:6 n3-d5	11.09 ± 0.02	4.0 ± 0.2	0.05	–	2.5	0.005	0.02	0.01
C20:1 n9	309.4	309.4	− 48	− 16	C20:0-d3	11.62 ± 0.01	3.4 ± 0.2	0.05	–	2.5	0.01	0.03	0.025
C20:0	311.2	311.2	− 44	− 20	C20:0-d3	12.04 ± 0.01	3.5 ± 0.2	0.05	–	2.5	–^c^	–^c^	0.05^e^
C21:0	325.2	325.2	− 48	− 16	C20:0-d3	12.25 ± 0.01	3.3 ± 0.4	0.075	–	2.5	0.05	0.2	0.075
C22:6 n3	327.4	327.4	− 46	− 16	C22:6 n3-d5^f^	9.09 ± 0.06	10.2 ± 0.6	0.05	–	2.5	0.025	0.08	0.05
	327.4	283.4	− 46	− 16		9.09 ± 0.07	9.3 ± 0.8	0.075	–	2.5	0.05	0.2	0.075
C22:5 n6	329.2	329.2	− 48	− 20	C22:6 n3-d5^f^	10.49 ± 0.02	4.9 ± 0.2	0.05	–	2.5	0.01	0.03	0.025
	329.5	285.2	− 48	− 20		10.49 ± 0.02	4.8 ± 0.4	0.05	–	2.5	0.025	0.08	0.05
C22:5 n3	329.2	329.2	− 48	− 20	C22:6 n3-d5^f^	10.02 ± 0.03	6.3 ± 0.2	0.05	–	2.5	0.01	0.03	0.025
	329.2	285.2	− 48	− 20		10.02 ± 0.04	6.3 ± 0.6	0.075	–	2.5	0.05	0.2	0.075
C22:4 n6	331.3	331.3	− 40	− 20	C22:6 n3-d5^f^	10.88 ± 0.02	4.1 ± 0.1	0.05	–	2.5	0.005	0.02	0.01
	331.3	287.3	− 40	− 20		10.88 ± 0.02	4.1 ± 0.3	0.075	–	2.5	0.05	0.2	0.075
C22:2 n6	335.3	335.3	− 46	− 20	C22:6 n3-d5	11.76 ± 0.01	3.2 ± 0.3	0.05	–	1	0.01	0.03	0.025
C22:1 n9	337.5	337.5	− 46	− 26	C20:0-d3	12.11 ± 0.01	3.1 ± 0.8	0.05	–	2.5	0.025	0.08	0.05
C22:0	339.2	339.2	− 46	− 20	C20:0-d3	12.45 ± 0.01	3.3 ± 0.6	0.075	–	2.5	0.05	0.2	0.075
C23:0	353.2	353.2	− 48	− 16	C20:0-d3	12.65 ± 0.01	4.4 ± 0.8	0.075	–	1.0	0.05	0.2	0.075
C24:1 n9	365.4	365.4	− 48	− 20	C20:0-d3	12.51 ± 0.01	4.0 ± 0.5	0.075	–	1.0	0.05	0.2	0.075
C24:0	367.4	367.4	− 46	− 20	C20:0-d3	12.84 ± 0.01	4.8 ± 0.9	0.25	–	2.5	0.1	0.4	0.25
**Internal standards**													
C8:0-d15	158.2	158.2	− 26	− 9	IS	3.28 ± 0.01	3.14 ± 0.1						
C12:0-d23	222.2	222.2	− 44	− 18	IS	5.08 ± 0.02	4.4 ± 0.2						
C16:0-d4	259.2	259.2	− 46	− 21	IS	10.35 ± 0.03	6.4 ± 0.4						
C18:2 n6-d4	283.2	283.2	− 52	− 20	IS	9.45 ± 0.06	10.4 ± 0.6						
C18:1 n9-d17	298.2	298.2	− 52	− 20	IS	10.72 ± 0.02	4.2 ± 0.2						
C18:0-d5	288.2	288.2	− 48	− 21	IS	11.44 ± 0.01	3.4 ± 0.3						
C20:5 n3-d5	306.2	306.2	− 44	− 20	IS	7.38 ± 0.04	7.4 ± 0.6						
	306.2	262.2	− 44	− 20		7.38 ± 0.04	6.6 ± 0.9						
C20:4 n6-d8	311.2	311.2	− 48	− 20	IS	9.29 ± 0.06	9.3 ± 0.4						
	311.2	267.2	− 48	− 22		9.28 ± 0.06	8.2 ± 1.7						
C20:3 n6-d6	311.5	311.5	− 46	− 17	IS	10.32 ± 0.03	5.3 ± 0.3						
C20:0-d3	314.2	314.2	− 50	− 21	IS	12.03 ± 0.01	3.6 ± 0.3						
C22:6 n3-d5	332.5	332.5	− 42	− 20	IS	9.01 ± 0.06	10.0 ± 0.8						
	332.5	288.2	− 42	− 24		9.01 ± 0.06	9.5 ± 1.3						

^a^Mean ± SD of the retention time in three different batches

^b^Mean ± SD of the full width at half maximum (FWHM) in three different batches

^c^No LOD can be determined because analyte is also detectable in blank injection

^d^No calibration possible due to high background levels

^e^Determined by at least 2× peak height of blank injection and accuracy of 80–120%

^f^For quantification of fatty acid using the transition based on decarboxylation, the [M-H-44]^−^ transition of the IS was used

^g^Quadratic regression

### Sample preparation

For quantification of fatty acyl concentrations in oils, 4–5 mg oil were diluted with 1.5 ml *iso*-propanol. One hundred microliters of this solution were mixed with 300 μl *iso*-propanol, 10 μl of antioxidant mixture (0.2 mg/ml butylated hydroxy toluene (BHT), 100 μM indomethacin, 100 μM trans-4-(-4-(3-adamantan-1-yl-ureido)-cyclohexyloxy)-benzoic acid (*t*-AUCB) in MeOH), 50 μl water and 100 μl 0.6 M KOH in MeOH/H_2_0 (75/25, *v*/v). Samples were hydrolyzed (30 min, 60 °C). Following neutralization with 20 μl 25% HAc samples were diluted (20 μl/500 μl) in EtOH and additionally 10 μl/100 μl for high-concentrated fatty acids and 50 μl/100 μl for low-concentrated fatty acids. 

For quantification of fatty acyls in plasma, 100 μl plasma were mixed with 10 μl antioxidant mixture and 400 μl ice-cold *iso*-propanol. Following centrifugation (4 °C, 20,000×*g*, 10 min), 450 μl of the supernatant were collected. For hydrolysis (30 min, 60 °C), 100 μl 0.6 M KOH in MeOH/H_2_0 (75/25, v/v) were added. After neutralization with 20 μl 25% HAc samples were diluted (20 μl/500 μl and subsequently 50 μl/100 μl) in EtOH. Free fatty acids in plasma were analyzed in the same way without hydrolysis and the following dilution: 10 μl/100 μl for high-concentrated fatty acids and 40 μl/100 μl for low-concentrated fatty acids.

For quantification of NEFA in plant oils solid-phase extraction on aminopropyl columns (1 ml volume, 100 mg bed weight, Supelco/Merck, Darmstadt, Germany) was used to remove triacylglycerols [13]. Approximately 10 mg of oils were diluted in 1 ml chloroform/*iso*-propanol 2/1 (*v*/*v*). Ten microliters of BHT (0.02 mg/ml in MeOH) and 10 μl C20:4 n6 (160 μM) as internal standard were added. The columns were washed with two cartridge volumes of diethyl ether/HAc 98/2 (*v*/*v*) and two cartridge volumes of chloroform/*iso*-propanol 2/1 (*v*/*v*). Samples were loaded onto the cartridges and triacylglycerols were removed with two cartridge volumes of chloroform/*iso*-propanol 2/1 (*v*/*v*). NEFA were eluted with two cartridge volumes of diethyl ether/HAc 98/2 (*v*/*v*). The eluate was neutralized with 1 ml 1 M NaHCO_3_, and the upper layer was collected and evaporated to dryness (vacuum concentrator, 30 °C, 1 mbar; Christ, Osterode, Germany). The residue was reconstituted in 200 μl EtOH, diluted 50 μl/100 μl for low-concentrated fatty acids as well as 10 μl/100 μl and subsequently 40 μl/100 μl for high-concentrated fatty acids.

## Results and discussion

A sensitive and selective quantification of fatty acids by means of LC-MS was developed: mass spectrometric detection was optimized and a rapid, efficient chromatographic separation was developed. The method performance was characterized and the results were compared with those from GC-FID analysis. Finally, the method was applied to the analysis of fatty acyls as well as NEFA in plasma and in plant oils.

### Optimization of mass spectrometric detection

**Fig. 1:**
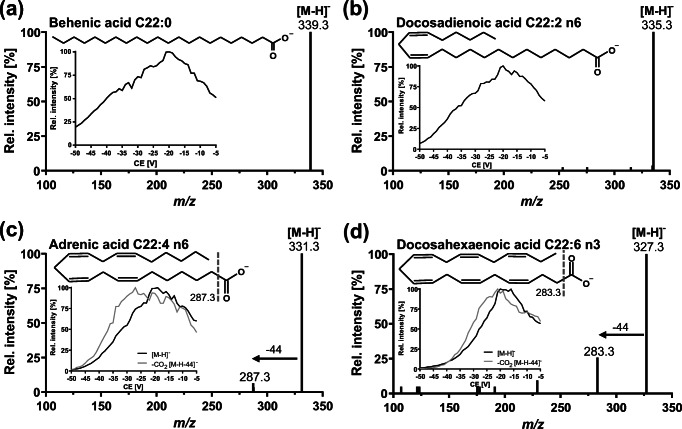
Collision-induced dissociation (CID) product spectra of [M-H]^−^ ions of selected fatty acids with 22 carbon atoms and increasing number of double bounds. **a** Behenic acid, **b** docosadienoic acid, **c** adrenic acid, and **d** docosahexaenoic acid. Insert: Optimization of collision energy (CE) for *pseudo*-SRM and decarboxylation. Ionization was carried out in negative electrospray ionization mode

Fatty acids contain a carboxy group; thus, ionization was carried out in ESI(−) mode. This leads to the formation of [M-H]^−^ ions which where the dominating ions detected in MS full-scan experiments. The declustering potential was optimized in single ion monitoring mode of the [M-H]^−^ ions for each fatty acid (Table 1). Monitoring of collision-induced dissociation (CID) fragment spectra revealed no fragmentation for fatty acids with ≤ 3 double bonds, while for PUFA with ≥ 4 double bonds the formation of [M-H-44]^−^ ions was observed, resulting from a decarboxylation (Fig. 1). The lack of detection of fragments of the linear hydrocarbon backbone is consistent with earlier reports [20]. Interestingly, for n6-PUFA, the intensity of the fragment resulted from CO_2_ loss was higher compared to n3-PUFA which might be due to the spatial proximity of the double bonds to the carboxyl group in n6-PUFA. This may lead to a higher fragmentation rate through stabilization of the fragment ion charge in the unsaturated carbon chain. *Pseudo* scheduled selected reaction monitoring mode (*pseudo*-SRM), i.e., isolating the *m*/*z* of [M-H]^−^ ions in Q1 and Q3, was used for quantification of fatty acids which do not show fragment ions in CID. Using the highest collision energy which did not lead to a decrease of the [M-H]^−^ ion in *pseudo*-SRM mode, co-eluting isobaric matrix is likely to be fragmented which increases the specificity of the detection. PUFA bearing ≥ 4 double bonds were detected in *pseudo*-SRM and additionally in regular SRM mode by using transition of the loss of CO_2_. However, because *pseudo*-SRM and decarboxylation are rather unspecific, chromatographic separation is crucial for isomeric fatty acids. The following critical isobaric separation pairs were identified among the biological occurring PUFA: C18:3 n3 (ALA) and n6 (GLA), C20:3 n6 (DGLA) and n9, C20:4 n3 (n3-ARA) and n6 (ARA), as well as C22:5 n3 (n3-DPA) and n6 (n6-DPA).

### Optimization of chromatographic separation

Liquid chromatographic separation of low to moderately polar substances, including fatty acids, is commonly achieved using a C18 column [16, 20, 21, 27]. Using a state-of-the-art sub-2 μm particle filled C18 reversed phase column (column 1; Zorbax Eclipse Plus, 150 × 2.1 mm, Agilent, Waldbronn, Germany) and a linear H_2_O/MeOH/ACN gradient (Table 2), retention times of fatty acids were unacceptably long. Even using an optimized gradient with a long isocratic step (11 min) at 100% B, long-chain fatty acids eluted at late retention times (C24:0 20.28 min). Moreover, ALA and GLA were not separated (*R* = 0.87). Different columns with lower hydrophobicity were tested aiming to increase the selectivity to separate isomers. ALA and GLA were the most critical separation pair; thus, a gradient was chosen yielding an optimal retention factor (*k*) and an isocratic step at their retention time (Table 2): In order to adjust the appropriate elution power (percentage B) for the isocratic step, it was calculated from the linear starting gradient at which %B ALA elutes and the isocratic step was set to this calculated percentage B minus 5%.

**Table 2:** Selection of LC-column for the chromatographic separation of fatty acids. Summarized are the stationary phases of the tested analytical columns and the column dimensions. The quality of the separation and the suitability of the method was characterized by the retention factor (*k*) and the full peak width at half maximum (FWHM) of the first eluting fatty acid to adjust the initial gradient conditions, the retention time of the last eluting fatty acid to define the total run time, and the chromatographic resolution of the isobaric analytes. ACN/MeOH/HAc (80/15/0.1; *v*/*v*/*v*) was used as the organic eluent (B) and the aqueous eluent (A) was 0.1% acetic acid with 5% B. The flow rate was 0.3 ml/min

**Column dimension**							**Isocratic step (%B)**	***k***_**C6:0**_	**FWHM**_**C6:0**_ **(s)**	**RT**_**C24:0**_ **(min)**	***R***_**GLA/ALA**_	***R***_**C20:3n9/n6**_	***R***_**n6/n3-ARA**_	***R***_**n6/n3-DPA**_
Stationary phase	Brand manufacturer	Length (mm)	Internal diameter (mm)	Particle size^a^ (μm)	Pore size^a^ (nm)	Carbon load^a^ (%)								
*Linear gradient*^b^														
C18, doubly endcapped	ZORBAX Eclipse Plus Agilent	150	2.1	1.8	9.5	9.0	Linear	5.63	7.6	–	1.18	2.63	2.21	3.39
Biphenylpropyl, multi-endcapping	Nucleoshell Macherey-Nagel	150	2.0	2.7 (core–shell)	9.0	5.2	Linear	3.41	10.6	28.30	0.57	3.07	2.25	3.63
Pentaflourophenyl, TMS endcapping	Kinetex Phenomenex	100	2.1	2.6 (core–shell)	10	9.0	Linear	3.19	16.7	23.50	0.51	2.29	1.75	2.73
Phenyl (ether linked), polar endcapping	Synergi Polar-RP Phenomenex	100	2.0	2.5	10	11	Linear	3.30	14.4	23.28	0.33	1.14	0.89	1.32
C8, hybrid silica, endcapping	Triart YMC	100	2.0	1.9	12	17	Linear	6.55	9.9	28.83	1.36	3.17	2.55	3.76
C8, TMS endcapping	Kinetex Phenomenex	100	2.1	2.6 (core–shell)	10	8.0	Linear	4.77	13.1	28.28	1.68	3.56	2.91	4.42
*Optimized gradient (isocratic step for GLA/ALA separation at indicated % B)*^c^														
C18, doubly endcapped	ZORBAX Eclipse Plus Agilent	150	2.1	1.8	9.5	9.0	90^d^	0.90	6.6	20.28	0.87	2.83	1.72	3.02
Biphenylpropyl, multi-endcapping	Nucleoshell Macherey-Nagel	150	2.0	2.7 (core–shell)	9.0	5.2	68	1.44	2.4	12.98	0.88	2.75	2.43	3.17
Pentaflourophenyl, TMS endcapping	Kinetex Phenomenex	100	2.1	2.6 (core–shell)	10	9.0	56^d^	2.56	2.4	11.74	0.65	2.30	1.94	2.67
Phenyl (ether linked), polar endcapping	Synergi Polar-RP Phenomenex	100	2.0	2.5	10	11	58^d^	2.10	3.9	11.77	0.46	0.92	1.11	1.14
C8, hybrid silica, endcapping	Triart YMC	100	2.0	1.9	12	17	71	2.36	2.4	13.26	1.25	2.76	3.35	3.69
C8, TMS endcapping	Kinetex Phenomenex	100	2.1	2.6 (core–shell)	10	8.0	66	2.36	2.4	12.96	1.88	2.89	3.21	3.68

^a^According to the manufacturer

^b^Gradient: 0–2 min 20% B, 2–26 min linear to 90% B, 26–27 min linear to 100% B, 27–31 min 100% B, 31–33 min linear to 20% B, 33–36 min reconditioning

^c^Gradient: 0–1 min 20% B, 1–1.5 min linear to the respective % B of the isocratic step, 1.5–8 min % B of the isocratic step, 8–11 min linear to 100% B, 11–14 min 100% B, 14–14.5 min linear to 20% B 14.5–15 min reconditioning

^d^Gradient: 0–1 min 50% B, 1–3 min linear to 90% B, 3–9 min 90% B, 9–11 min linear to 100% B, 11–22 min 100% B, 22–23.5 min linear to 50% B, 23.5–25 min reconditioning

^d^Initial gradient condition was A/B 90/10

With a biphenyl stationary phase (column 2; Nucleoshell, 150 × 2.0 mm, 2.7 μm particle size (core–shell), Macherey-Nagel, Düren, Germany) sufficient separation of ALA and GLA (*R* = 0.88) could not be achieved. It seems that the *π*–*π* interactions between the isolated double bonds of the fatty acids and the aromatic double bonds of the biphenyl phase do not provide sufficient selectivity. This is supported by the results from a second biphenyl phase (Raptor Biphenyl, 100 × 2.1 mm, 2.7 μm core–shell particle), Restek, Bad Homburg vor der Höhe, Germany). On this column with a considerably lower hydrophobicity, a separation of ALA and GLA was not possible (*R* = 0.95), despite the shorter length comparable to that of column 2. Modification of the aromatic ring structure to pentafluorophenyl residues (column 3; PFP Kinetex, 100 × 2.1 mm, 2.6 μm core–shell particle) or linkage of a phenyl moiety to the silica gel via an ether bridge (column 4; Synergi Polar-RP, 100 × 2.0 mm, 2.5 μm particle size, both Phenomenex, Aschaffenburg, Germany) led to poor separation of ALA and GLA (*R* = 0.65 and 0.46, respectively).

A baseline separation of ALA and GLA (*R* > 1.5) could be achieved on a C8 reversed phase column with dimensions of 100 × 2.1 mm, 2.6 μm core–shell particle (Kinetex, Phenomenex, Aschaffenburg, Germany). Increasing the hydrophobicity by ethyl-bridged hybrid silica (column 5, Triart, 100 × 2.0 mm, 1.9 μm particle size, YMC, Dinslaken, Germany) failed to further improve the separation (*R*_ALA/GLA_ = 1.25).

**Fig. 2:**
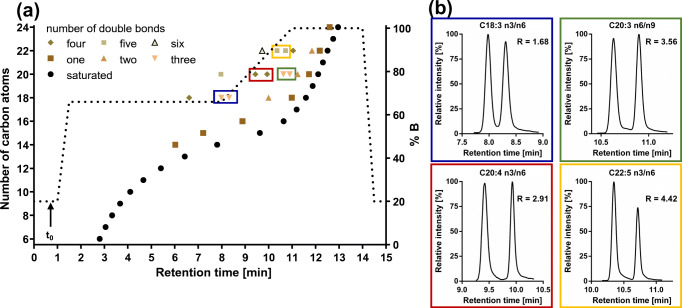
**a** Relationship between retention time of the fatty acids and the number of carbon atoms as well as double bounds. **b** Chromatographic separation for isomeric fatty acids. Separation was carried out on an RP-8 column (2.1 × 100 mm, 2.6 μm core–shell particle, pore size 10 nm) with (**a**) a H_2_O/ACN/MeOH/HAc gradient with a flow rate of 0.3 ml/min. The void volume was approx. 0.24 ml (0.8 min)

With the optimized chromatographic conditions on the Kinetex C8 column, 41 fatty acids and 11 internal standards could be separated within 13.5 min (Fig. 2, ESM Fig. S2). The saturated fatty acids eluted over the entire run time, while the retention times for unsaturated fatty acids depended on the number of double bonds (Fig. 2). Hu et al. and Bromke et al. also described a pronounced relationship between retention time, number of carbon atoms, and number of double bonds [20, 21]. C24:0 eluted last with a retention time of 12.96 min. In order to remove potentially retained non-polar matrix the isocratic step at 100% B was held for one void volume (0.24 ml, 0.8 min). Including re-equilibration the final run time was 15 min with highly stable retention times showing a variation (relative standard deviation, RSD) of < 0.20% or < 0.02 min for intra-batch (*n* = 24) and < 0.75% or < 0.07 min for inter-batch (three batches, *n* = 30; Table 1).

**Fig. 3:**
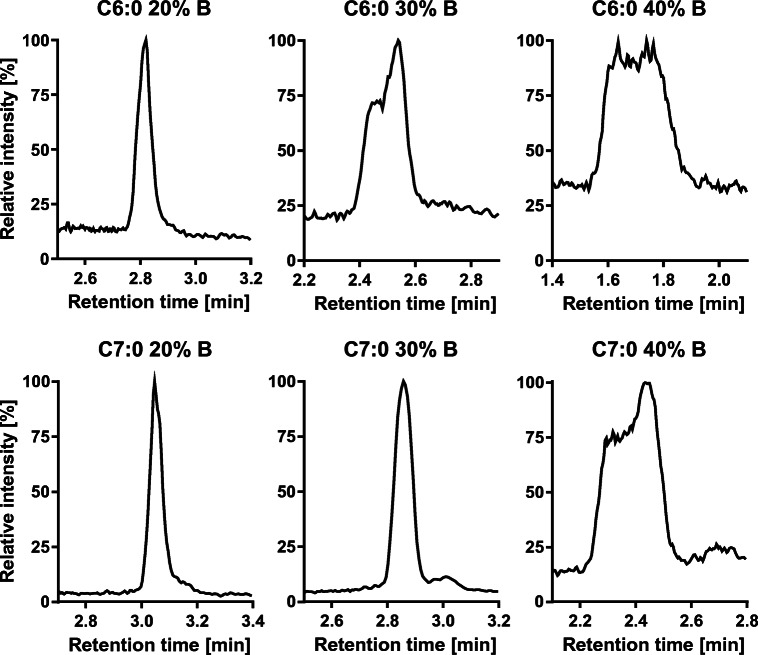
Effect of pre-concentration step in the gradient on peak shapes of the first eluting fatty acids. Shown are injections (10 μl) of a fatty acid standard (0.5 μM) in ethanol at different initial gradient conditions. The initial conditions were held for 1 min, then the % B was increased to 66% B in 0.5 min

Regarding the start conditions of the gradient, it turned out that a pre-concentration step [28] with low elution power (20% B) is required for a good separation of early eluting fatty acids. The lipophilic nature of fatty acids makes the use of the more non-polar injections solvent EtOH necessary; otherwise, long-chain fatty acids are discriminated (ESM Fig. S3). Without the pre-concentration step, the strong elution power of the injection solvent deteriorates the peak shape of the early eluting analytes (Fig. 3). With a retention factor *k* > 1, the analytes are well separated from void volume (*k* = 2.36 for C6; Table 2) using 20% B for the initial step. ALA and GLA are separated by a long isocratic step at 66% B (6.5 min, *R* = 1.88), other critical separation pairs such as n3- and n6-DPA were separated within a linear solvent gradient. Interestingly, while C20:3 n9 and n6 were easily separable (*R* = 2.89), C20:3 n6 and n3 as well as C18:1 n9 and n7 could not be separated on any of the tested columns. It is consistent with literature that RP-LC does not allow to separate C20:3 n6 and n3 [16, 22]. However, in our experience, C20:3 n3 and C18:1 n7 do not occur or only occur at low concentrations in biological samples [7, 10, 29]. Thus, the quantification of C20:3 n6 and C18:1 n9 in sum with the respective isomer does not seem problematic for a correct determination of the quantitative fatty acid pattern in cells, blood, tissues and the most edible oils.

The method described herein is superior compared to previous LC-MS approaches. It requires no derivatization as used by several other groups [14, 15, 23, 24], which shortens sample preparation considerably. The total run time of 15 min allows rapid analysis. Even though a run time of around 15 min is also achieved in other methods, these methods quantify a considerably lower number of analytes (23 fatty acids [25], 14 fatty acids [14], 30 fatty acids [22]).

### Sensitivity

The limit of detection (LOD) and lower limit of quantification (LLOQ) was determined according to the Guideline on Bioanalytical Method Validation of the European Medicines Agency (EMA) [30]. The LOD was set to the lowest injected standard yielding a signal-to-noise ratio (S/N) of ≥ 3 and the LLOQ was set to the lowest standard yielding a signal-to-noise ratio of ≥ 5 and an accuracy of 80–120% within the calibration curve. The LODs of the fatty acids detected by *pseudo*-SRM ranged mainly between 5 and 25 nM (0.01–0.08 ng on column; Table 1), whereas the LODs for the transition based on decarboxylation were higher (25–50 nM; 0.08–0.2 ng on column) due to the low intensity of the [M-H-44]^−^ fragment. Therefore, fatty acids bearing ≥ 4 double bounds were also quantified in *pseudo*-SRM mode. The sensitivity is consistent with earlier reported LODs, e.g., 0.02–0.1 μM (0.05–0.32 ng on column) [25] and 0.03–0.3 μM (0.1–1 ng on column) [22]. It should be noted that we used a 20-year-old middle class mass spectrometer. With state-of-the-art high-resolution MS instruments such as LTQ Orbitrap Elite [16] or highly sensitive QqQ MS QTRAP 5500 [20], LODs of 1–2 orders of magnitude lower can be achieved.

The group of Hu et al. found a low LOD also for C16:0 and C18:0 (0.05 ng/ml; 0.20 nM and 0.18 nM, respectively) [20]. In our hands, blank injections and even LC-MS measurements without injection also showed peaks for C16:0 and C18:0 (ESM Figs. S4 and S5) which could not be completely reduced by using pure solvents, glassware instead of plastic and methanol as well as *iso*-propanol for washing the injection system between runs. High background signals of these fatty acids—used ubiquitously in consumer products such as plastic ware—were also described by other groups [18, 23]. For these analytes, we set the LLOQ to the concentration yielding a peak height of at least twofold of the peak height in blank injections and an accuracy within the calibration curve of 80–120% (Table 1) which was 0.5 μM for C16:0 and 1 μM for C18:0. The ULOQ was set to 20 μM. By using a deuterated internal standard for each of these compounds (C16:0-d4 and C18:0-d5) ion suppression occurring at this high concentration could be compensated allowing a linear regression. (ESM Figs. S4 and S6). In order to enable simultaneous quantification of C18:1 n9, which is a main FA in biological samples, quadratic least square regression (weighting: 1/*x*^2^) was used. Only low carry-over was observed in the preceding injection of a high-concentrated standard (ESM Fig. S5). For quantification of all other fatty acids, linear calibration up to 2.5 μM was used. This strategy using fatty acid-specific concentration ranges of the calibration series (Table 1) allows the rapid quantification of fatty acids in biological samples with only one set of calibrators. Due to the use of a large number of isotopically labeled IS, the analysis is also robust and shows high accuracy and precision.

### Accuracy and precision

**Fig. 4:**
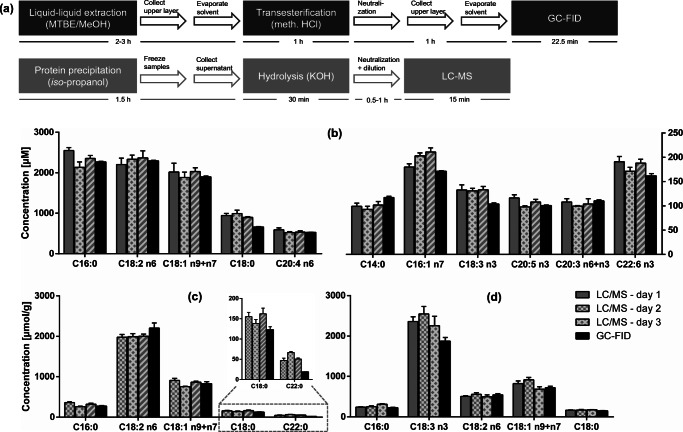
Accuracy and precision of the method: The intra- and inter-day variability of fatty acyl concentration as well as comparison of the LC-MS method to quantification by GC-FID in human plasma and edible oils are shown. **a** The sample preparation. Fatty acyl concentrations in **b** human plasma, **c** sunflower oil, and **d** flaxseed oil determined on 3 days by means of LC-MS (mean ± SD, *n* = 3) compared to the concentrations determined by means of GC-FID (mean ± SD, *n* = 3)

The accuracy of the developed analytical LC-MS method was assessed by comparing the fatty acyl concentrations in plasma and plant oils with those obtained by a validated GC-FID analysis (Fig. 4) which can be considered the gold standard of fatty acid analysis. The use of GC-FID provides an orthogonal quantification which is not dependent on standard concentrations due to the mass-sensitive detector allowing to deduce absolute concentration based on one reference compound. Sample preparation for gas chromatographic determination included lipid extraction with methanol/methyl *tert*-butyl ether (MTBE) and transesterification to FAME [12], whereas for LC analysis, the samples were diluted with *iso*-propanol and the supernatant after centrifugation was directly hydrolyzed (Fig. 4a) [31]. The major fatty acyls quantified in the plasma were C16:0, C18:2 n6, and C18:1 n9 + n7, followed by C18:0 and C20:4 n6 as previously described for healthy subjects [7, 32]. The main n3-PUFA were ALA, EPA, and DHA having concentrations of around 150 μM (Fig. 4b). Given the difference in sample preparation, both methods showed an excellent match of the determined concentrations (agreement 80–120%, Fig. 4b). Only for C18:0, slightly higher concentrations are obtained by means of LC-MS, presumably because of its high background signal. Differently diluted hydrolyzed plasma samples show that the matrix leads only to low or no ion suppression allowing robust quantification of the fatty acyls in biological samples (ESM Fig. S7).

In plant oils, similar levels of fatty acyls were found following quantification by means of GC-FID or LC-MS (Fig. 4c, d). For the main fatty acyls in flaxseed oil or sunflower oil, the concentration agreement was also good (70–130%, Fig. 4c, d). Therefore, we conclude that the simple sample preparation by dilution with *iso*-propanol and saponification is suitable for the quantification of fatty acyls in protein-rich matrices as well as in fatty matrices. It should be noted that more fatty acids could be quantified by LC-MS than by GC-FID due to the higher sensitivity of the LC-MS method. The LLOQ of the LC-MS measurement is ≤ 75 nM for almost all fatty acids, whereas it is more than one order of magnitude higher for the GC-FID analysis. This made it possible to quantify for example C22:5 n6 in plasma or C22:1 n9 in flaxseed oil allowing to gain a more comprehensive picture of the fatty acid pattern of biological samples and plant oils. It should be noted that GC-(MS) following transesterification on the one hand allows the simultaneous detection of a more comprehensive set of fatty acids compared to LC-MS and on the other hand electron ionization MS provides more structural information based on fragments. For example, Lisa et al. found 81 fatty acyls in animal fats including also branched and *trans*-isomers [33]. However, if only major fatty acids are of interests as it is in numerous studies of modern life science, LC-MS is in our hands the method of choice because of the rapid sample preparation and analysis.

In order to evaluate the precision of the analytical procedure, human plasma samples as well as two edible oils with different fatty acid pattern were analyzed on three separate days (inter-day variance Fig. 4, *n* = 3). The intra-day variability was assessed by calculating the RSD on each single day (*n* = 3; ESM Table S1). Both parameters were lower than 15% for almost all fatty acyls in plasma and plant oils and thus meet the criteria required by the EMA guideline [30] demonstrating a high precision of the developed method. Only long-chain saturated and monounsaturated fatty acids such as C20:0 and C20:1 n9 showed in part higher variations. Interestingly, the results using transitions based on decarboxylation resulted in higher RSD, e.g., 15% for C22:5 n3 vs. 5% in *pseudo*-SRM, which might be explained by low intensity of the [M-H-44]^−^ fragment and thus low peak heights. Therefore, quantification should be carried out by *pseudo*-SRM mode and the second transition can additionally be used for confirmation.

The determination of fatty acyls by LC-MS offers a much faster and more sensitive method than GC-FID analysis. We could show that the method leads to consistent and precise results. The easy and rapid sample preparation via direct saponification in *iso*-propanol is not only fast but allows the simultaneous total oxylipin determination (ESM Table S2) [31]. Thus, our approach makes it possible to analyze both oxidized fatty acyls and their precursors from a single sample preparation.

### Non-esterified fatty acids in plasma and edible oils

**Fig. 5:**
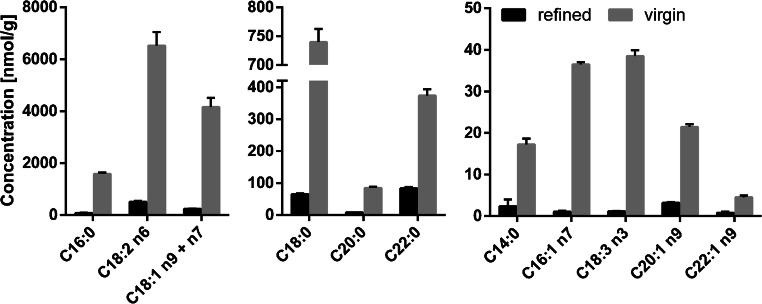
Concentration of non-esterified fatty acids in virgin and refined sunflower oil determined by LC-MS; 10 mg sunflower oil were dissolved in chloroform/*iso*-propanol (2/1, *v*/*v*) and triacylglycerols were removed by solid-phase extraction on aminopropyl cartridges [13]. Refined and virgin oils were obtained from a local supermarket and analyzed in triplicate (mean ± SD)

Concentrations of NEFA were determined in plasma (ESM Table S3) and virgin, cold-pressed sunflower oil as well as refined sunflower oil (Fig. 5) using the described LC-MS method. In plasma, NEFA could be directly analyzed after dilution of the sample with *iso*-propanol. The obtained concentrations (ESM Table S3) were in good agreement with the results described by other groups [16, 17]. The accuracy of the measurement was supported by the standard addition procedure using ARA resulting in a concentration of 2.0 μM in plasma, compared to 2.1 ± 0.1 μM by external calibration (ESM Fig. S8). This underlines the robustness of the method against matrix effects and demonstrates that the method allows the reliable quantification of three parameters from a single sample preparation: quantification of fatty acyls, NEFA and total oxylipins.

For quantification of NEFA in plant oils, the excess of triacylglycerols was reduced by SPE using aminopropyl cartridges as described [13, 34]. As expected, the concentrations of NEFA were considerably lower in refined sunflower oil than in virgin sunflower oil, since the NEFA are removed in the deacidification step of the refining process [35]. C18:2 n6 was the fatty acid with the highest concentration, followed by C18:1 n9 + n7, C16:0, and C18:0. Thus, the concentrations of NEFA represent the total fatty acid distribution (Fig. 4c). Because of the sensitivity of the method, we could detect low-concentrated fatty acids such as C22:1 n9, which often could not be reported [36, 37]. Due to the direct analysis of the NEFA by LC-MS, our method does not require derivatization for gas chromatographic analysis compared to previous methods, where different derivatization strategies such as esterification [38], silylation [36] or dimethylamidation [39] are used. The targeted approach allows quantification of NEFA even in the presence of triacylglycerols that may not have been completely removed by the SPE. Thus, the presented LC-MS method herein allows the reliable quantification of NEFA in biological samples, which is of pivotal importance for the characterization of both edible oils [35, 40] as well as biological samples such as plasma [2, 41].

## Conclusion

A new LC-MS method for the quantification of fatty acids in biological samples was developed. Using an optimized C8 reversed phase column, 41 fatty acids and 11 isotopically labeled fatty acids as internal standards could be separated within a total run time of only 15 min. Despite using a rather old, middle class QqQ MS, the method is sensitive with a LLOQ of 10–75 nM for most fatty acids. The low inter-day and inter-operator variability of < 20% indicates a high precision of the method. The concentrations of fatty acyls determined by LC-MS in plasma and plant oils are consistent with those of a gas chromatographic analysis ensuring accurate and comparable results by the developed method. A major strength of the approach is the rapid sample preparation by hydrolysis and dilution allowing high sample throughput. Moreover, the analysis can be combined with the analysis of PUFA oxidation products (eicosanoids and other oxylipins) [31]. Finally, LC-MS analysis allows to quantify NEFA in presence of triacylglycerols which is of pivotal importance for the analysis of biological samples such as plant oils.

## Supplementary information

ESM 1 (PDF 923 kb)

## Abbreviations

ACN: Acetonitrile
ALA: α-Linolenic acid
ARA: Arachidonic acid
BHT: Butylated hydroxy toluene
CID: Collision-induced dissociation
DGLA: Dihomo-γ-linolenic acid
DHA: Docosahexaenoic acid
DPA: Docosapentaenoic acid
EPA: Eicosapentaenoic acid
EMA: European Medicines Agency
EtOH: Ethanol
FWHM: Full width at half maximum
FAME: Fatty acid methyl ester
GC-FID: Gas chromatography-flame ionization detection
GLA: γ-Linolenic acid
HAc: Acetic acid
IS: Internal standard
LA: Linoleic acid
LC-MS: Liquid chromatography-mass spectrometry
LOD: Limit of detection
LLOQ: Lower limit of quantification
MeOH: Methanol
MTBE: Methyl *tert*-butyl ether
NEFA: Non-esterified fatty acid
PUFA: Polyunsaturated fatty acid
R: Resolution
RSD: Relative standard deviation
RT: Retention time
SPE: Solid phase extraction
SRM: Scheduled selected reaction monitoring
