# Supplementary material for: Rapid quantification of fatty acids in plant oils and biological samples by LC-MS
Source: Anal Bioanal Chem. 2021 Jul 22;413(21):5439–51. doi: 10.1007/s00216-021-03525-y (PMC8405509; doi:10.1007/s00216-021-03525-y)
Supplement: Supplementary file 1 — (PDF 923 kb) [file 216_2021_3525_MOESM1_ESM.pdf]

Supplementary Information

Rapid quantification of fatty acids in plant oils and biological samples by LC-MS

Elisabeth Koch, Michelle Wiebel, Carolin Hopmann, Nadja Kampschulte and Nils Helge Schebb*

Chair of Food Chemistry, Faculty of Mathematics and Natural Sciences, University of Wuppertal, Wuppertal, Germany

*Contact information of the corresponding author:

Nils Helge Schebb
Chair of Food Chemistry
Faculty of Mathematics and Natural Sciences
University of Wuppertal
Gaussstrasse 20
42119 Wuppertal
nils@schebb-web.de
Tel: +49-202-439-3457

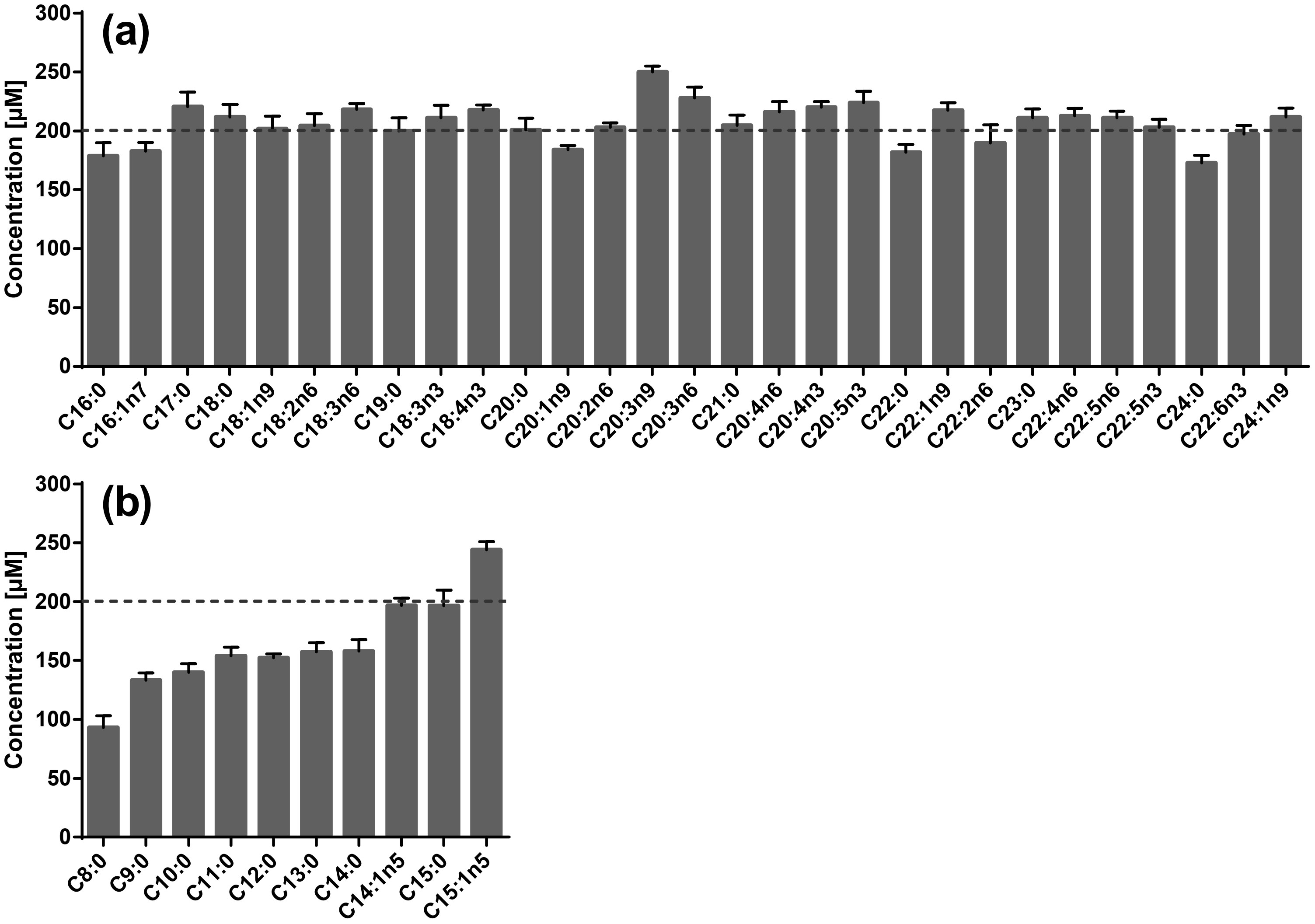

Fig. S1: Concentrations of fatty acids in calibration stock solutions determined by GC-FID (mean ± SD, n = 3). (**a**) 50 µl of the calibration stock solutions were evaporated to dryness, reconstituted in *n*-hexane and transesterified to methyl esters with methanolic HCl as described [1]. C25:0 FAME was used as internal standard. (**b**) 50 µl of the calibration stock solutions were evaporated to dryness and reconstituted in methyl *tert*-butyl ether. 50 µl trimethylsilyl sulfonium hydroxide (TMSH) was added and the samples were directly analyzed by GC-FID. C25:0 FAME was used as internal standard. Dashed lines indicate the nominal concentration of 200 µM. It should be noted that saturated fatty acids having ≤ 14 carbon atoms could not quantitively be transesterified to FAME neither by methanolic HCl nor by TMSH.

[1] Ostermann AI, Muller M, Willenberg I, Schebb NH (2014) Determining the fatty acid composition in plasma and tissues as fatty acid methyl esters using gas chromatography - a comparison of different derivatization and extraction procedures. Prostag Leukotr Ess 2014;91(6):235-41.

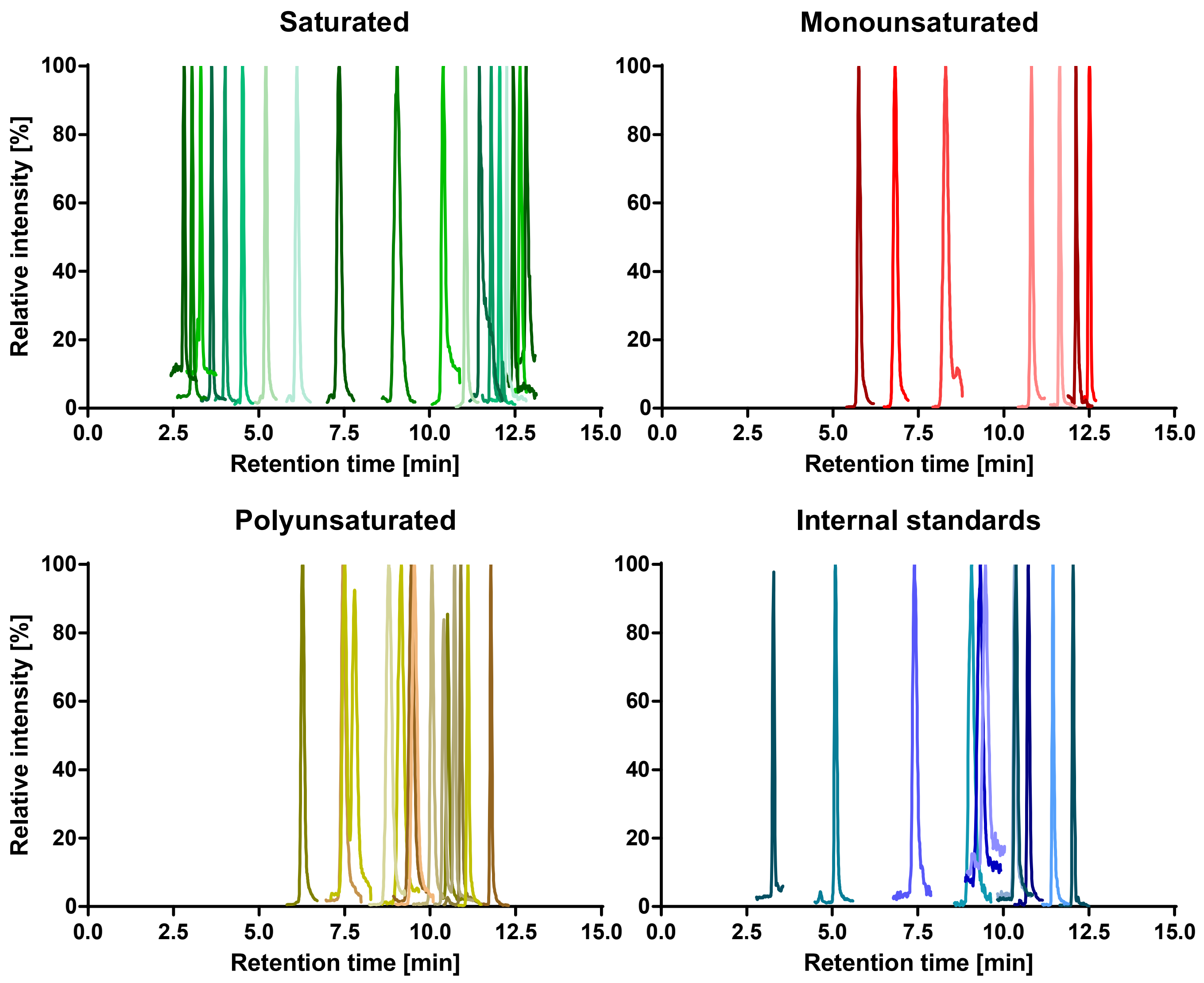

Fig. S2: Chromatographic separation of 41 fatty acids and 11 fatty acid internal standards. Shown are relative intensities of the *pseudo*-SRM transitions of the fatty acids after injection of 10 µl of fatty acid standard solutions (1 µM). Separation was carried out on RP-8 column (2.1 × 100 mm, particle size 2.6 µm (core-shell), pore size 10 nm) with a H_2O/ACN/MeOH/HAc gradient. Fatty acids are grouped according to their number of double bounds.

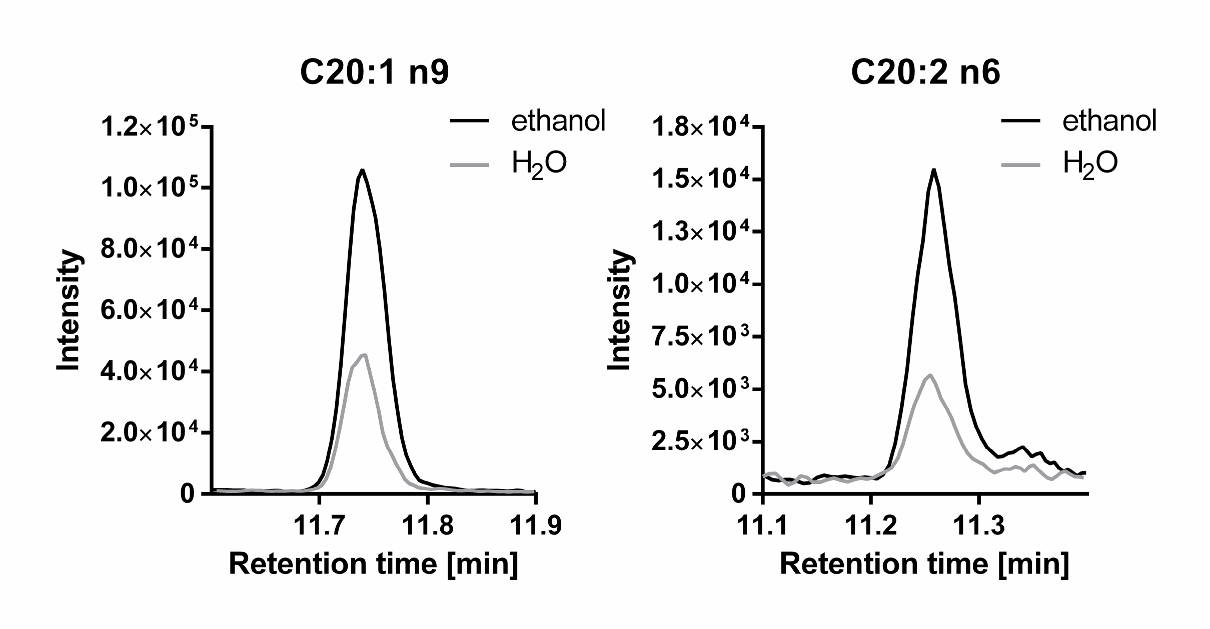

Fig. S3: Peak intensity of C20:1 n9 and C20:2 n6 in hydrolyzed rapeseed oil diluted in water or ethanol. Rapeseed oil was diluted in *iso*-propanol and hydrolyzed with 0.6 M KOH. The hydrolysate was diluted and injected (10 µl) in either water or in ethanol. Shown are exemplarily the *pseudo*-SRM signals of two long-chain fatty acids.

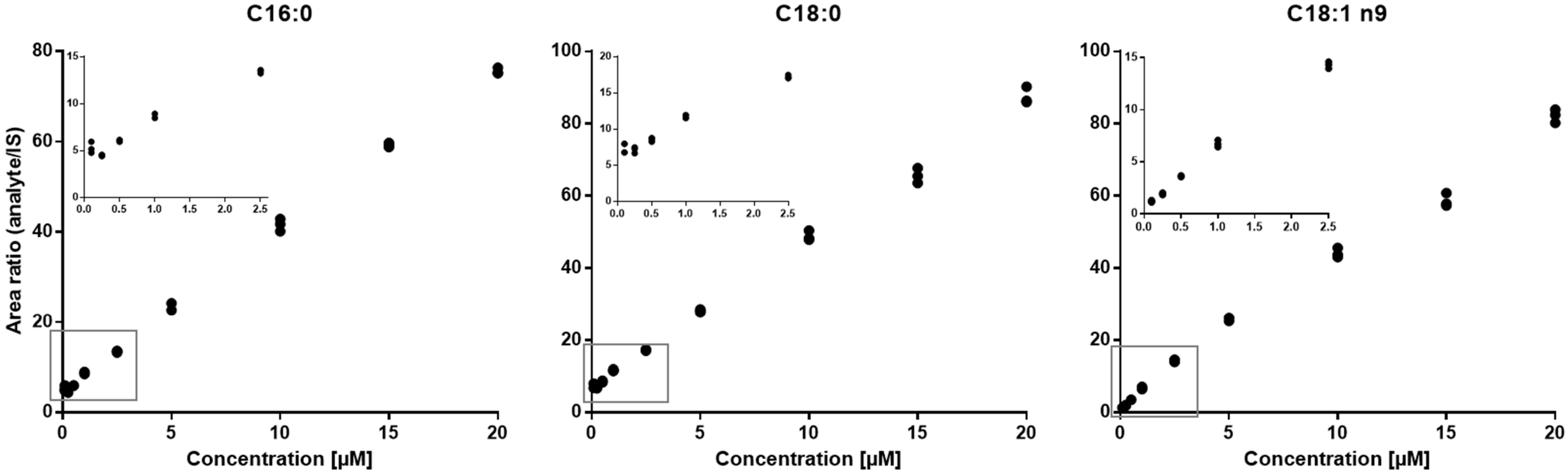

Fig. S4: Background levels of ubiquitously detectable fatty acids and their calibration curves. Area ratios (triple injections) are plotted against the concentration of the calibration standard. C16:0 and C18:0 also showed high peaks in blank injections and low concentrated calibration standards resulting in higher LLOQs. The LLOQ was set to the concentration yielding a peak height of at least twofold of the peak height in blank injections and an accuracy within the calibration curve of 80-120%. For C16:0 and C18:0 a linear regression could be used up to 20 µM and for C18:1 n9 quadratic regression up to 15 µM was applied.

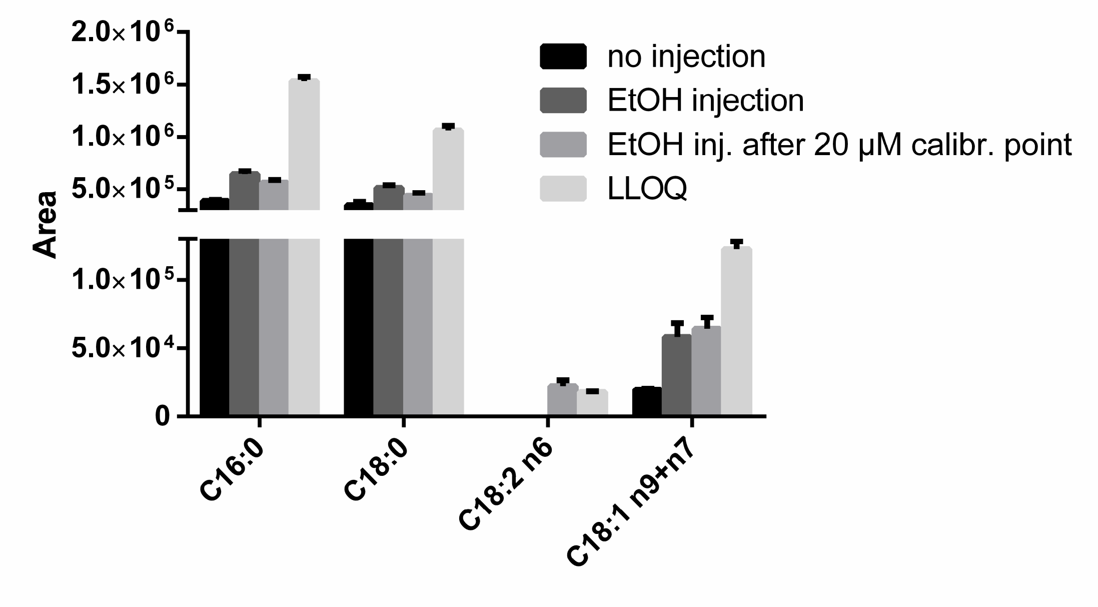

Fig. S5: Origin of background signals of ubiquitously detectable fatty acids. Shown are areas (mean ± SD, n = 3) of the fatty acids in LC-MS measurements without injection, injecting only pure ethanol as well as injection of pure ethanol after analysis of the highest calibration point (20 µM).

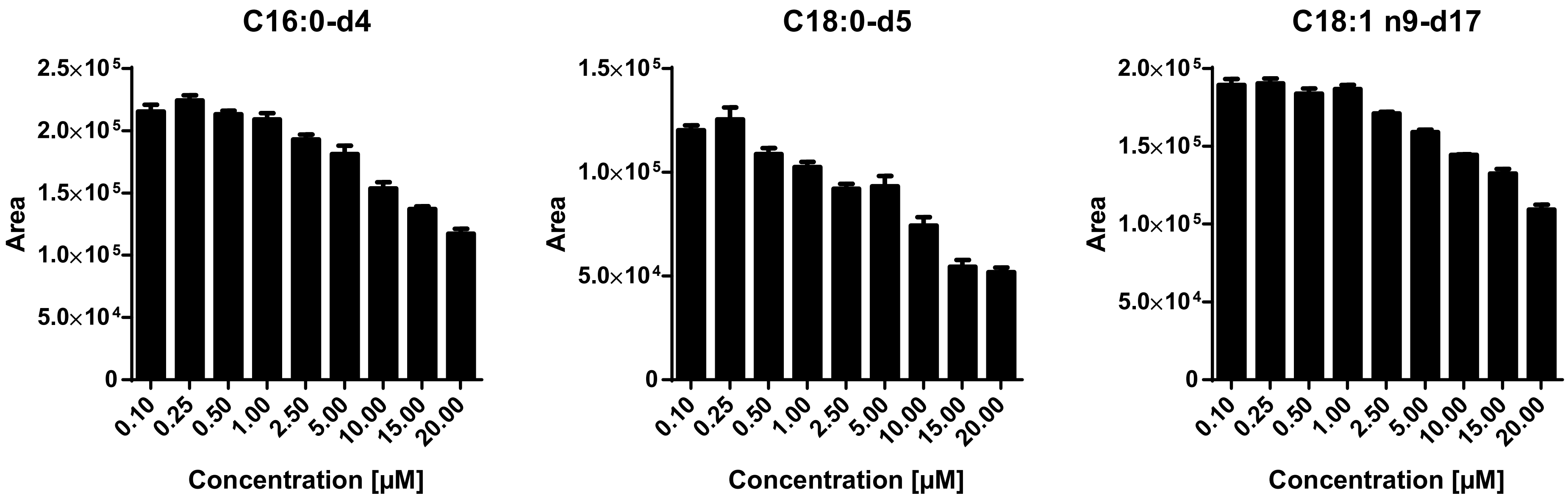

Fig. S6: Ion suppression of the internal standards C16:0-d4, C18:0-d5 and C18:1 n9-d17 (0.2 µM) with increasing fatty acid concentration in the calibrants. Areas (mean ± SD, n = 3) are plotted against the concentration in the calibration standard. The decreasing areas of the internal standards with increasing fatty acid concentrations indicates ion suppression.

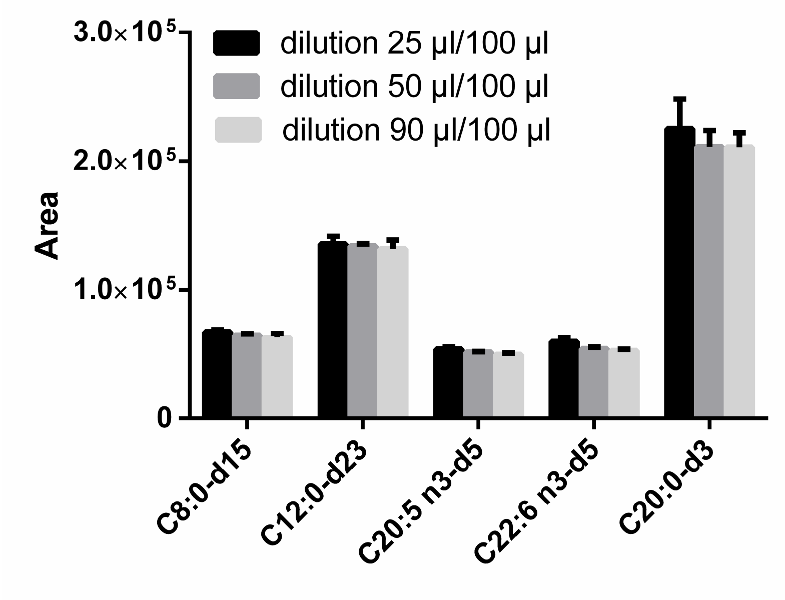

Fig. S7: Influence of dilution of human plasma samples on the areas of internal standards. Shown are areas (mean ± SD, n = 4) of internal standards in sequentially diluted hydrolyzed human plasma samples: 20 µl hydrolysate/500 µl ethanol. Subsequent dilutions were: high: 25 µl/100 µl; medium: 50 µl/100 µl; low: 90 µl/100 µl.

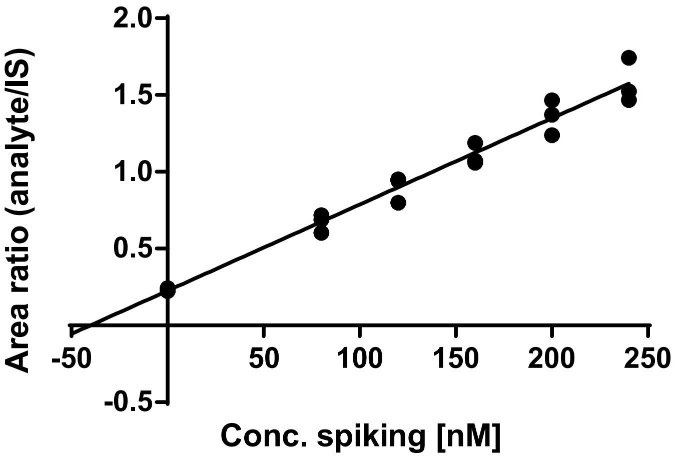

Fig. S8: Evaluation of accuracy of the determination of non-esterified fatty acids in human plasma using the standard addition procedure. Arachidonic acid was spiked at different levels in human plasma. 200 µl *iso*-propanol was added to 50 µl of plasma and 10 µl of the supernatant was diluted with ethanol (final volume: 100 µl). The x-intercept was determined using linear regression and had a best-fit value of -40.2 nM. The concentration in the vial of ARA in the non-spiked human plasma using the external concentration with internal standard was found to be 41.8 nM ± 1.9 nM (mean ± SD, n = 3).

	Human plasma		Sunflower oil		Flaxseed oil	
	Intra-day RSD	Inter-day RSD	Intra-day RSD	Inter-day RSD	Intra-day RSD	Inter-day RSD
C14:1 n5	6.8	11				
C14:0	7.0	7.4				
C16:1 n7	4.1	8.1	1.4	20	7.6	8.6
C16:0	3.2	8.1	6.4	15	3.1	13
C18:4 n3	7.2	6.6				
C18:3 n6	5.5	4.8				
C18:3 n3	5.1	5.5	5.1	10	4.9	8.6
C18:2 n6	7.2	6.9	2.6	2.9	2.8	7.8
C18:1 n9+n7	4.6	7.9	2.5	8.7	7.9	14
C18:0	2.2	6.6	8.6	10	3.7	6.2
C20:5 n3	4.8	8.4				
C20:5 n3 [1]	4.6	8.3				
C20:4 n6	6.3	8.2				
C20:4 n6 [1]	3.6	9.5				
C20:4 n3	8.9	7.7				
C20:3 n9	6.1	15				
C20:3 n6+n3	10	7.5			11	12
C20:2 n6	4.7	10				
C20:1 n9	4.9	21	7.7	17	6.2	8.9
C20:0	14	18	6.9	10	7.7	13
C22:6 n3	4.2	6.4				
C22:6 n3 [1]	8.1	8.7				
C22:5 n3	4.6	7.7				
C22:5 n3 [1]	15	13				
C22:5 n6	5.7	19				
C22:5 n6 [1]	11	17				
C22:4 n6	3.9	16				
C22:1 n9	12	35			6.6	17
C22:0			4.9	18		

[1] The $[M\text{-}H\text{-}44]^-$ transition was used for quantification.

Tab. S1: Intra- and inter-day precision of the fatty acyl concentrations in human plasma, sunflower oil and flaxseed oil. Fatty acyl concentrations were determined in triplicate on three separate days by means of LC-MS following hydrolysis. Intra- and inter-day variability was assessed by calculating the relative standard deviation on each single day and on all three days, respectively.

Precursor fatty acid	Analyte	Concentration [nM]
Oleic acid (C18:1 n9)	9(10)-Ep-stearic acid	90 ± 10
	9,10-DiH-stearic acid	22 ± 2
Linoleic acid (C18:2 n6)	9-HODE	1140 ± 30
	10-HODE	12.3 ± 0.5
	12-HODE	6.9 ± 0.5
	13-HODE	1930 ± 70
	15-HODE	16.4 ± 0.9
	9(10)-EpOME	95 ± 10
	12(13)-EpOME	78 ± 9
	9,10-DiHOME	18.7 ± 0.6
	12,13-DiHOME	6.4 ± 0.3
Linolenic acid (C18:3 n3)	9-HOTrE	26 ± 1
	13-HOTrE	60 ± 3
	9(10)-EpODE	4.3 ± 0.6
	12(13)-EpODE	2.6 ± 0.2
	15(16)-EpODE	30 ± 2
	9,10-DiHODE	0.75 ± 0.03
	12,13-DiHODE	0.49 ± 0.02
	15,16-DiHODE	23 ± 1
Mead acid (C20:3 n9)	5-HETrE	4.1 ± 0.2
Dihomo-γ-linolenic acid (C20:3 n6)	8-HETrE	21 ± 2
	12-HETrE	53 ± 2
	15-HETrE	36 ± 1
	14(15)-EpEDE	2.9 ± 0.4
Arachidonic acid (C20:4 n6)	5-HETE	94 ± 3
	8-HETE	123 ± 10
	9-HETE	240 ± 10
	11-HETE	240 ± 15
	12-HETE	200 ± 15
	15-HETE	163 ± 8
	16-HETE	1.5 ± 0.2
	17-HETE	0.35 ± 0.05
	18-HETE	0.75 ± 0.10
	20-HETE	1.46 ± 0.07
	12-HHTrE	2.4 ± 0.2
	tetranor-12-HETE	0.89 ± 0.04
	5(*S*),15(*S*)-DiHETE	9.48 ± 0.07
	8(*S*),15(*S*)-DiHETE	76 ± 2
	6-*trans*-LTB_4	3.7 ± 0.1
	6-*trans*-12-*epi*-LTB_4	7.1 ± 0.2
	8(9)-EpETrE	16 ± 2
	11(12)-EpETrE	18 ± 2
	14(15)-EpETrE	28 ± 3
	5,6-DiHETrE	13.8 ± 0.5
	8,9-DiHETrE	3.2 ± 0.2
	11,12-DiHETrE	1.07 ± 0.07
	14,15-DiHETrE	0.86 ± 0.05
	PGB_2	1.8 ± 0.2
	5(*R*,*S*)-F_{2t}-IsoP	0.47 ± 0.05
	20-COOH-ARA	6.6 ± 0.3

Eicosapentaenoic acid (C20:5 n3)	5-HEPE	30	±	2
	8-HEPE	46	±	3
	9-HEPE	117	±	6
	11-HEPE	52	±	3
	12-HEPE	116	±	7
	15-HEPE	102	±	6
	18-HEPE	104	±	5
	20-HEPE	1.7	±	0.2
	8(9)-EpETE	3.5	±	0.4
	11(12)-EpETE	3.1	±	0.4
	14(15)-EpETE	3.8	±	0.4
	17(18)-EpETE	6.0	±	0.7
	5,6-DiHETE	2.0	±	0.2
	8,9-DiHETE	0.43	±	0.01
	14,15-DiHETE	0.13	±	0.02
	17,18-DiHETE	0.72	±	0.03
Docosahexaenoic acid (C22:6 n3)	4-HDHA	45	±	2
	7-HDHA	49	±	2
	8-HDHA	74	±	3
	10-HDHA	61	±	5
	11-HDHA	108	±	4
	13-HDHA	69	±	5
	14-HDHA	82	±	6
	16-HDHA	73	±	4
	17-HDHA	79	±	4
	20-HDHA	97	±	6
	7(8)-EpDPE	6.3	±	0.7
	10(11)-EpDPE	5.4	±	0.6
	13(14)-EpDPE	5.3	±	0.7
	16(17)-EpDPE	5.2	±	0.7
	19(20)-EpDPE	9.7	±	0.9
	7,8-DiHDPE	2.8	±	0.1
	10,11-DiHDPE	0.56	±	0.04
	13,14-DiHDPE	0.45	±	0.02
	16,17-DiHDPE	1.26	±	0.04
	19,20-DiHDPE	2.79	±	0.07

Tab. S2: Concentration of total oxylipins in human plasma. 100 µl of human plasma were diluted with *iso*-propanol. The supernatant after centrifugation was hydrolyzed with potassium hydroxide, neutralized and loaded onto pre-conditioned solid phase extraction (SPE) cartridges (C8/anion exchange). The eluate after SPE was evaporated, reconstituted and analyzed by LC-MS (mean ± SD, n = 4) [2].

[2] Koch E, Mainka M, Dalle C, Ostermann AI, Rund KM, Kutzner L, Froehlich LF, Bertrand-Michel J, Gladine C, Schebb NH (2020) Stability of oxylipins during plasma generation and long-term storage. Talanta 2020;217.

Fatty acid	Concentration [µM]
C10:0	1.2 ± 0.2
C12:0	4.4 ± 0.5
C14:1 n5	1.7 ± 0.1
C14:0	15 ± 1
C15:0	1.4 ± 0.1
C16:1 n7	20 ± 1
C16:0	140 ± 10
C17:0	3.4 ± 0.2
C18:4 n3	0.12 ± 0.01
C18:3 n6	0.82 ± 0.09
C18:3 n3	12 ± 1
C18:2 n6	49 ± 5
C18:1 n9/7	190 ± 20
C18:0	46 ± 4
C20:5 n3	0.64 ± 0.07
C20:4 n6	2.7 ± 0.3
C20:4 n3	0.124 ± 0.005
C20:3 n9	0.06 ± 0.03
C20:3 n6/3	0.73 ± 0.07
C20:2 n6	0.70 ± 0.06
C20:1 n9	4.1 ± 0.3
C20:0	0.26 ± 0.05
C22:6 n3	2.9 ± 0.2
C22:5 n3	0.83 ± 0.07
C22:4 n6	0.34 ± 0.04
C22:1 n9	0.23 ± 0.07

Tab. S3: Concentration of non-esterified fatty acids in human plasma. 100 µl of human plasma were diluted with *iso*-propanol. 10 µl or 40 µl of the supernatant were diluted in ethanol (10 µl/100 µl for high concentrated fatty acids and 40 µl/100 µl for low concentrated fatty acids; mean ± SD, n = 3).
